# Supplementary material for: Broad-spectrum activity against mosquito-borne flaviviruses achieved by a targeted protein degradation mechanism
Source: Nat Commun. 2024 Jun 19;15:5179. doi: 10.1038/s41467-024-49161-9 (PMC11187112; doi:10.1038/s41467-024-49161-9)
Supplement: Supplementary file 1 — Supplementary Information [file 41467_2024_49161_MOESM1_ESM.pdf]

Supplementary Information

Broad-spectrum activity against mosquito-borne flaviviruses achieved by a targeted protein degradation mechanism

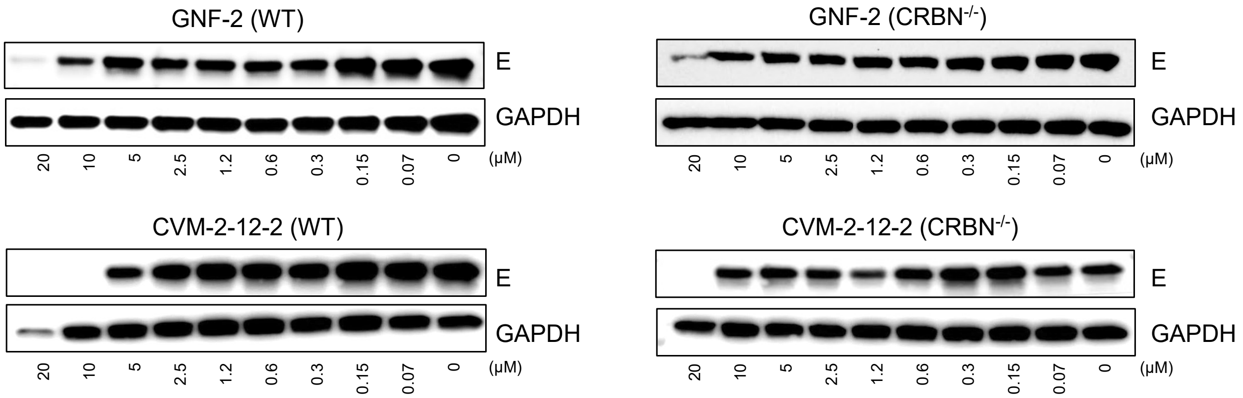

Supplementary Figure 1. Characterization of E abundance in dengue virus (DENV)-infected cells treated with E inhibitors GNF-2, and 2-12-2 (here labeled "CVM-2-12-2"). The cells are infected with the virus at a multiplicity of infection (MOI) of 1 for 1 hr. The infected cells were then treated with the inhibitors at the indicated concentrations starting at one hour post-infection and continuing to 24 hours post-infection. At that time, cell lysates were harvested and analyzed by Western blot. The representative results are shown from *n* = 3 independent experiments for GNF-2 and n=2 for 2-12-2.

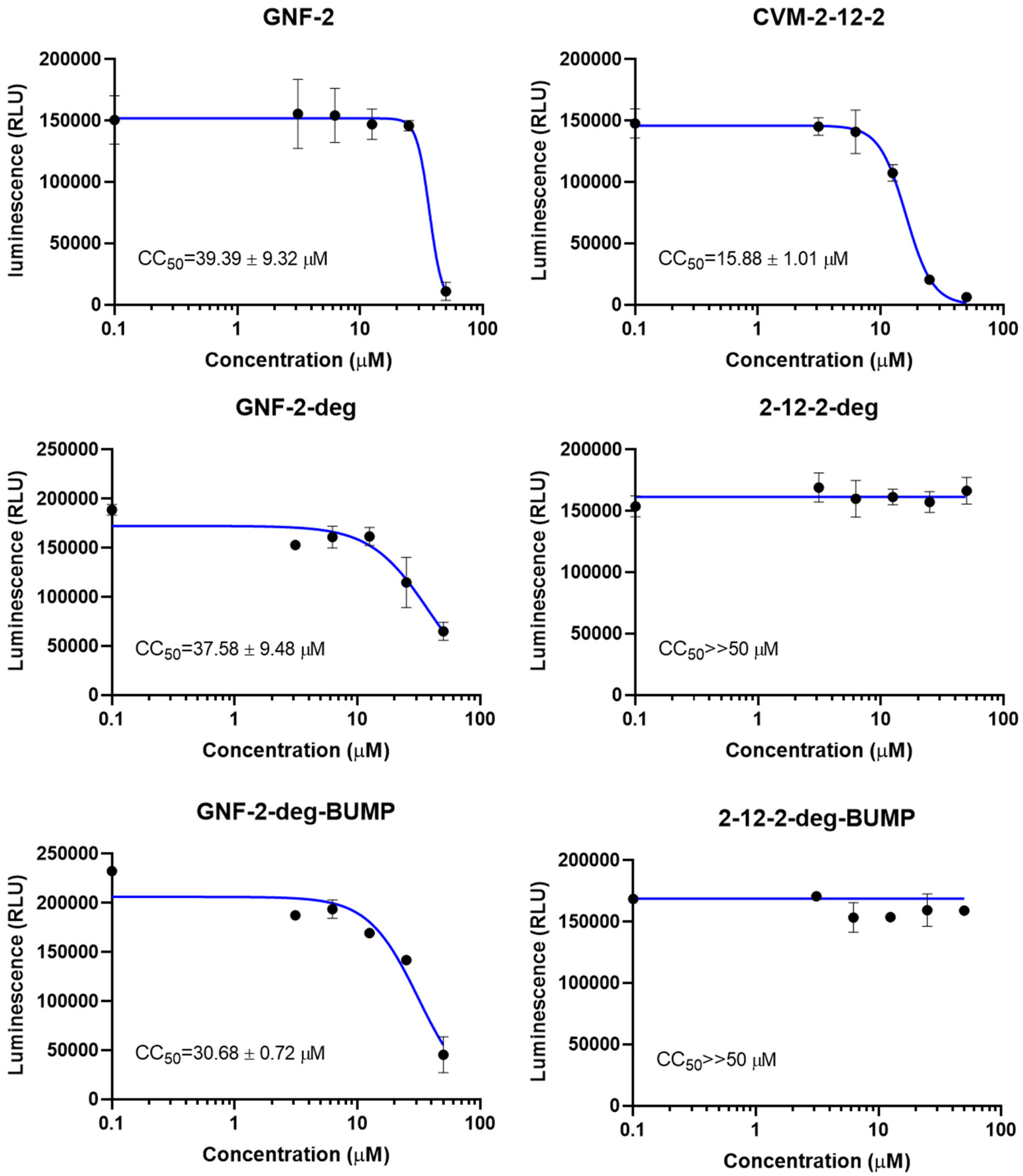

Supplementary Figure 2. The E degraders GNF-2-deg and 2-12-2-deg show insignificant cytotoxicity at the concentrations ranges at which they exhibit on-target E degradation and potent antiviral activity. Huh7.5 cells were treated with compounds at variable concentrations for 24 hr. Cell viability was then measured and nonlinear regression was used to determine the CC_{50} value, corresponding to the concentration of compound causing a 50% loss of viability. The representative results are from *n* = 2 independent experiments. The data are presented as mean ± standard deviation.

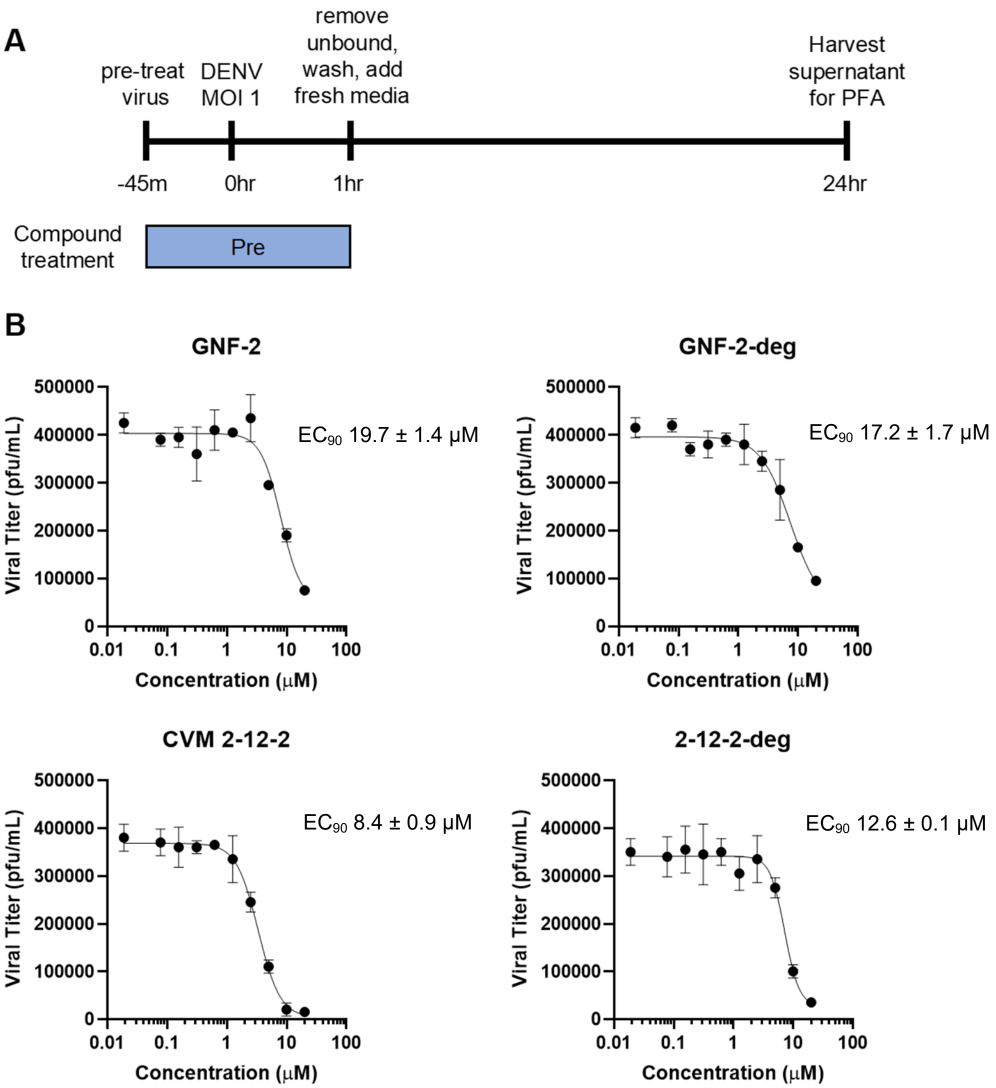

Supplementary Figure 3. Antiviral activities of E degraders GNF-2-deg and 2-12-2-deg and E inhibitors GNF-2 and 2-12-2 (here labeled "CVM 2-12-2") were compared in an infectivity assay in which compound treatment was limited to a preincubation of the viral inoculum and the initial 1 hour infection and culture supernatants were harvested at 24 hours post-infection to allow quantification of viral yield after approximately a single-cycle of infection. **(A)** Schematic of the infectivity assay. **(B)** Viral yield was quantified by viral plaque formation assay (PFA). Antiviral EC_{90} values were determined by nonlinear regression of the data and correspond to the concentration of the compound causing a 90% reduction in infectious particles. Representative results shown are from *n* = 2 independent experiments. The data are presented as mean ± standard deviation.

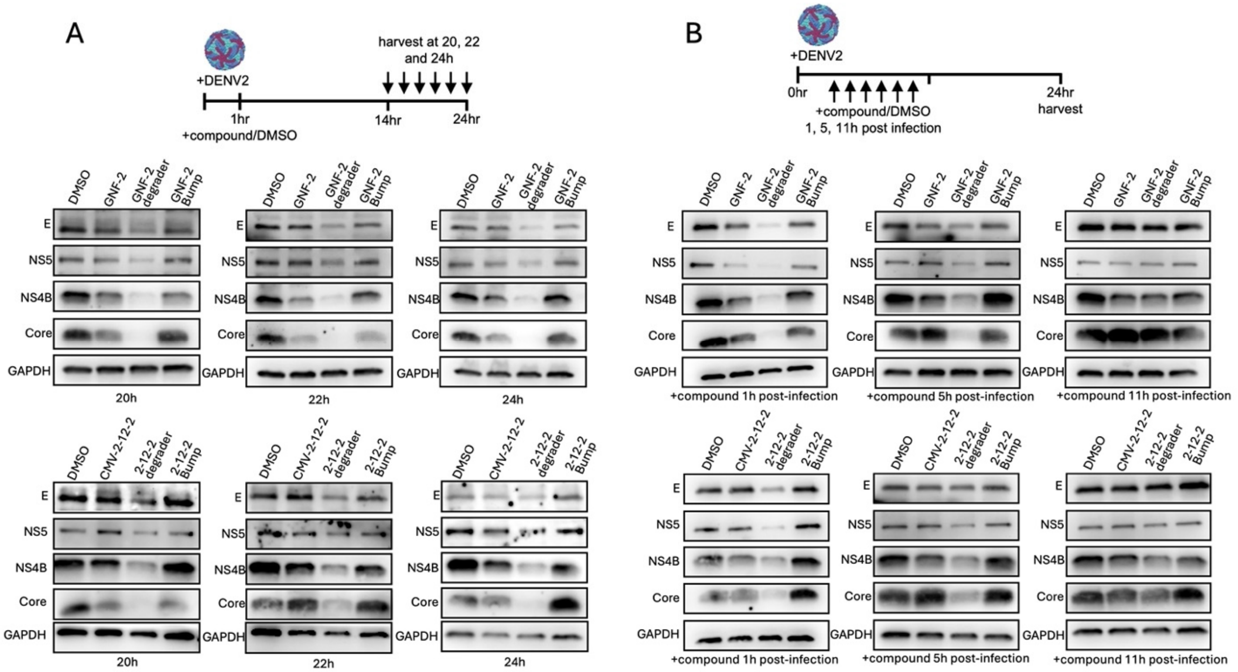

Supplementary Figure 4. E degraders GNF-2-deg and 2-12-2-deg cause depletion of other viral proteins in DENV-infected cells. **(A)** Cells were infected with DENV2 at an MOI of 1, and compounds were added to a final concentration of 2.5 μM at 1 hour post-infection (hpi). Cell lysates were harvested at 14 to 24-hpi for analysis of E, NS5, NS4B, and core by Western blot. Western blots show time-dependent depletion of E, NS5, NS4B, and core in the presence of GNF-2-deg and 2-12-2-deg at 20, 22, and 24hpi. Depletion of viral proteins is not observed in the presence of the parental inhibitors, GNF-2 and 2-12-2, or in the presence of negative control compounds GNF-2-deg-BUMP or 2-12-2-deg-BUMP. Timepoints earlier than 20 hours did not result in consistent detection of viral proteins (data not shown). The representative results are shown from *n* = 2 independent experiments. **(B)** Cells were infected with DENV2 at an MOI of 1, and compound treatments were initiated at 1 to 11 hpi at a concentration of 2.5 μM. Cell lysates were harvested at 24 hpi. Western blots show time-dependent depletion of E, NS5, NS4B, and core in the presence GNF-2-deg and 2-12-2-deg. Representative results are shown from *n* = 2 independent experiments. Antibodies used are as follows: GAPDH (GeneTex 6C5 GTX28245), E (4G2), core (Invitrogen GT574), NS4B (GeneTex GTX124250) and NS5 (GeneTex GT353). Schematics in this figure were created with Biorender.com and are published here under a CC-BY-NC-ND license. Open access to this article does not include the use of the images created in BioRender.

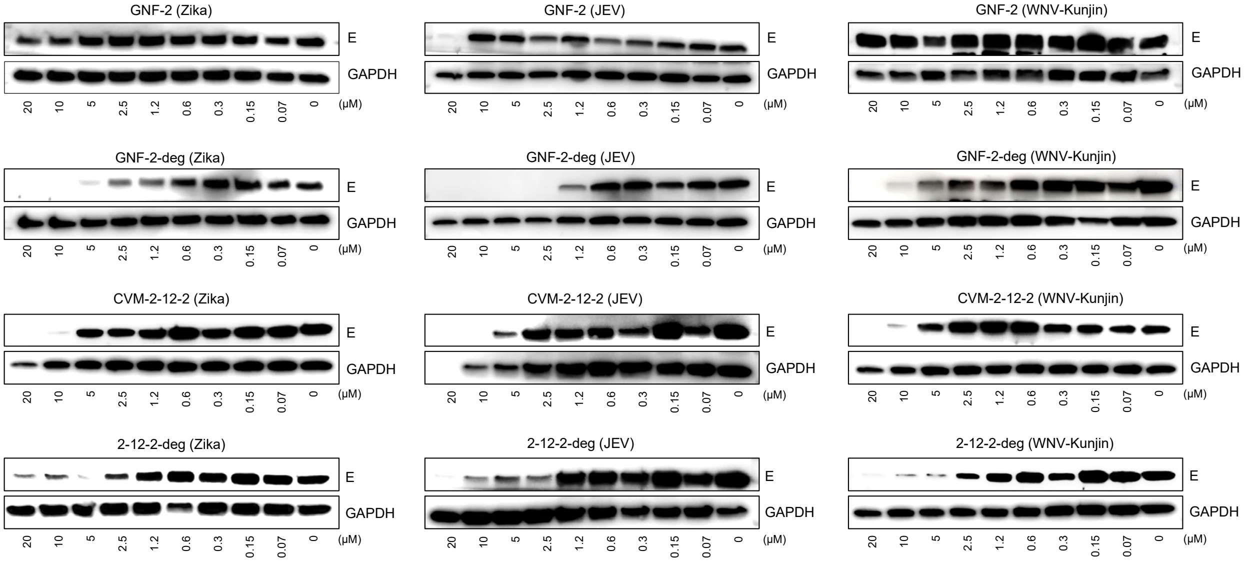

Supplementary Figure 5. DENV E degraders GNF-2-deg and 2-12-2-deg reduce intracellular E for multiple flaviviruses compared to parental inhibitors GNF-2 and 2-12-2. Cells were infected with the indicated virus at MOI of 1 for 1 hr, then washed and treated with the indicated compound. At 24 hours post-infection, cell lysates were harvested to analyze intracellular E by immunoblot (this figure), and culture supernatants were harvested to allow quantification of viral yield by PFA (Figure 5). Representative results are shown from *n* = 2 independent experiments.

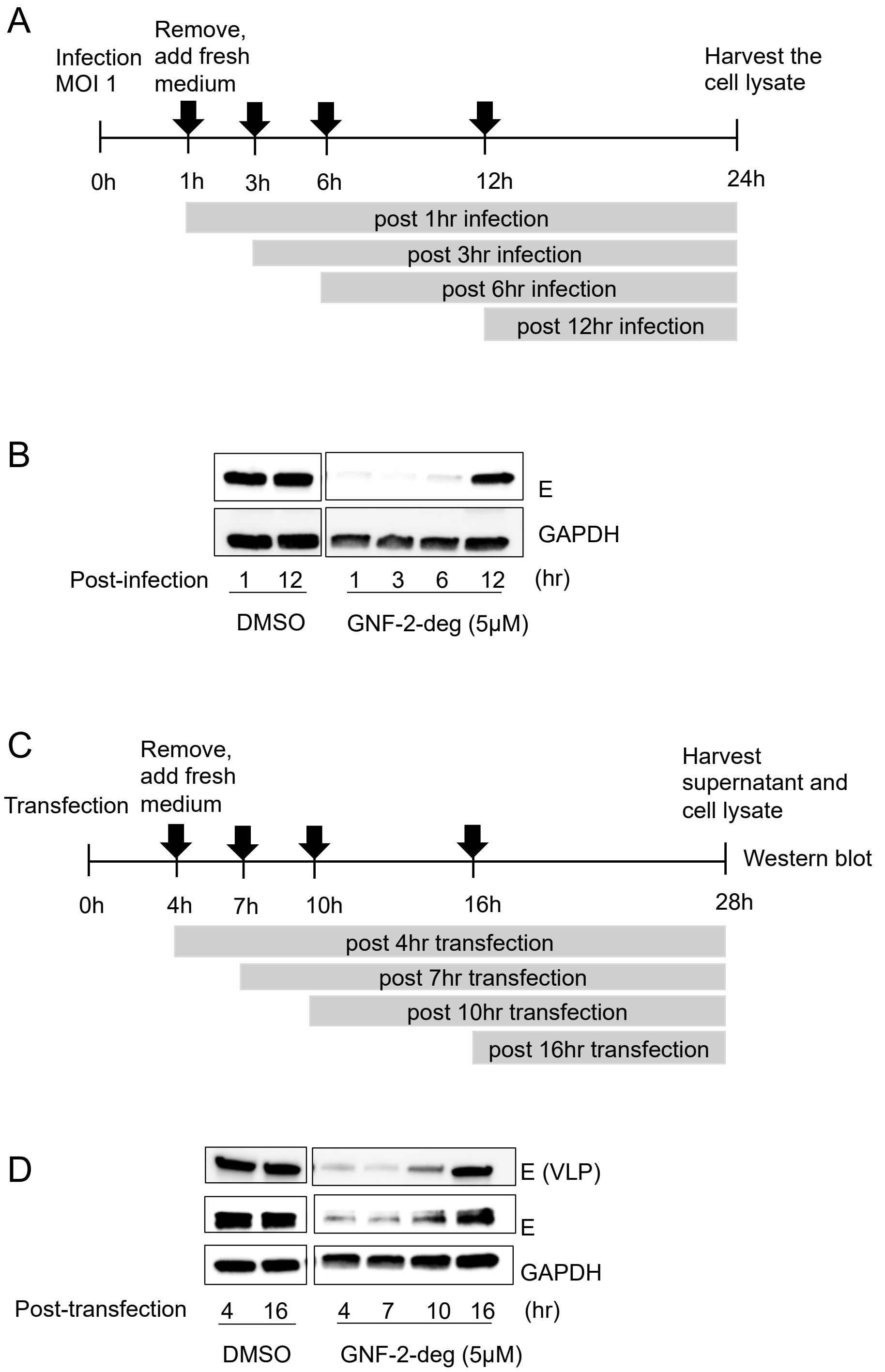

Supplementary Figure 6. (A) Schematic of time of addition assay. **(B)** Huh7.5 cells were infected at an MOI of 1, and GNF-2-deg was added to a final concentration of 5 μM at 1, 3, 6, and 12 hours post-infection (hpi). Cell lysates were collected for Western blot analysis at 24 hours post-infection. Depletion of E is not observed if addition of GNF-2-deg is delayed until 12 hpi. Representative results are shown from *n* = 2 independent

experiments. **(C)** Schematic of virus-like particle (VLP) time of addition assay. **(D)** Huh7.5 cells were transfected with the VLP prM-E plasmid, and GNF-2-deg was added to a final concentration of 5 μM at 4, 7, 10 and 16 hours post-transfection. Culture supernatants and cell lysates were collected for Western blot analysis at 28 hours post-transfection. The depletion of intracellular E and reduction in secreted VLPs are not observed if addition of GNF-2-deg is delayed until 16 hours post-transfection. Representative results are shown from *n* = 2 independent experiments.

Supplementary Note 1. Two hypotheses for depletion of core, NS4B, and NS5 in the presence of GNF-2-deg and 2-12-2-deg.

We can think of at least two non-mutually exclusive explanations for the effects of the E degraders on core, NS4B, and NS5 that are consistent with the E-specific and CRBN-dependent activity of GNF-2-deg and 2-12-2-deg in the VLP and live virus assays. First is that the 24 hour post-infection (hpi) time point at which we made these measurements is late enough for reinfection to have begun to occur. Reduced viral yield from the initial cycle of infection reduces the rate of reinfection, with add-on effects on viral translation and genome replication due to decreased template that affect the abundance of all of the viral proteins. In addition, the inhibitory effect of both the E inhibitors and the E degraders on fusion during this second cycle of infection would be expected to have an effect on all of the viral proteins as time extends beyond the initial cycle of infection. Any antiviral activity exerted through an off-target effect would also eventually lead to a decrease in all of the viral proteins. We will refer to these potential causes as "general antiviral activity" since they are not caused directly by targeted protein degradation induced by the E degraders acting on core, NS4B, or NS5. The second explanation we considered is that the E degraders may be targeting E within the context of the polyprotein, which could lead to degradation of core, NS4B, and NS5.

Since our original experiments examined compound treatment over the 1-24 hpi timeframe, we analyzed earlier time points to exclude the mechanisms of general antiviral activity outlined above. Our reasoning was that if GNF2-deg and 2-12-2-deg target E that has already been cleaved from the polyprotein, then we should be able to observe its depletion earlier than we can detect depletion of the other viral proteins brought about by general antiviral activity affecting a second round of infection. For these experiments, we chose a single concentration of 2.5 μM, which approximates the antiviral EC_{90} values of GNF-2-deg and 2-12-2-deg (3.5 μM and 1.7 μM, respectively) measured at an MOI of 1 and 1-24 hpi treatment (data in **Figure 3** in the manuscript). We observed depletion of core, NS4B, and NS5 alongside E at 20 and 22 hpi. Representative data from these experiments are shown **Supplementary Figure 4A**. We were unable to obtain meaningful data at time points earlier than this because we could not consistently detect the viral proteins by Western blot at 18 hpi or earlier, even when we increased the MOI to 5. While it is possible that a second round of infection is occurring at 20 hpi and contributing to the template pool for viral translation, the magnitude of this effect seems unlikely to account for the significant reductions in core, NS4B, and NS5 that we observe.

While our observations are consistent with possible targeting of the polyprotein by GNF-2-deg and 2-12-2-deg, more experiments are needed to test this hypothesis rigorously.

Primer	Sequence
E-F193L-FW	5’-AACCGGCCTGGATTTAAACGAGATGGTGCT-3’
E-F193L-RV	5’-AGCACCATCTCGTTTAAATCCAGGCCGGTT-3’
E-M196V-FW	5’- GGATTTCAACGAGGTGGTGCTGCTGC -3’
E-M196V-RV	5’- GCAGCAGCACCACCTCGTTGAAATCC -3’
E-F279S-FW	5’-CGGCAACCTGCTGAGCACCGGCCATTTG-3’
E-F279S-RV	5’-CAAATGGCCGGTGCTCAGCAGGTTGCCG-3’
E-F193L/M196V-FW	5’-CCGGCCTGGATTTAAACGAGGTGGTGCTGC-3’
E-F193L/M196V-RV	5’-GCAGCACCACCTCGTTTAAATCCAGGCCGG-3’

Supplementary Table 1. List of oligonucleotides used in the study for site-directed mutagenesis of the VLP plasmid.
